# Successful Management of a Giant Pituitary Lactosomatotroph Adenoma Only with Cabergoline

**DOI:** 10.1155/2013/134241

**Published:** 2013-05-09

**Authors:** Emre Bozkirli, Okan Bakiner, Emine Duygu Ersozlu Bozkirli, Eda Ertorer, Neslihan Bascil Tutuncu, Nilgun Guvener Demirag

**Affiliations:** ^1^Department of Endocrinology and Metabolism Diseases, Faculty of Medicine, Baskent University, Turkey; ^2^Department of Internal Medicine, Faculty of Medicine, Baskent University, Turkey

## Abstract

Although advances in endocrinologic and neuroradiologic research allow easier recognition of pituitary adenomas, giant pituitary tumours are relatively rare. In the literature, the term “giant” is generally used when a pituitary tumour becomes larger than 4 cm in diameter. Cabergoline is a potent and long-acting inhibitor of prolactin secretion, which exhibits high specificity and affinity for dopamine D2 receptor. Herein, we report a 46-year-old woman with a giant lactosomatotroph pituitary adenoma, sized 6 × 5 × 5.5 cm, who is treated successfully only with cabergoline. The patient showed dramatic response to cabergoline treatment by means of clinical, biochemical and radiological imaging findings. Cabergoline seems to be safe and effective in the treatment of prolactin and growth hormone cosecreting pituitary adenomas as well as prolactinomas. However, surgical or more aggressive approach must be considered where indicated.

## 1. Introduction

Pituitary adenomas are classified as “giant” when their diameter exceeds 4 cm [1]. The most common histological subtype of giant pituitary adenomas is macroprolactinomas. More than 70% of prolactinomas occur in females, whereas macroadenomas are detected more frequently among men [2]. Additionally, macroadenomas occur less frequently than microadenomas [3].

Giant pituitary tumors are very rare and usually invade the surrounding structures. These tumors are very difficult to treat and can behave aggressively [4, 5]. Unless urgent surgery is needed, medical therapy with a dopamine agonist such as bromocriptine or cabergoline can be the first line treatment [6, 7].

Cabergoline is a synthetic ergoline that exhibits high specificity and affinity for dopamine D2 receptor. It is a potent and long-lasting dopamine agonist, which has an elimination half-life, ranging between 63 and 109 hours [8]. When compared with bromocriptine, many studies pointed out the better efficacy and tolerance of cabergoline [9–11]. It is also a potent drug for treating acromegaly, even for patients with normoprolactinemia [12].

Herein, we describe the case of a woman with an extremely large pituitary adenoma cosecreting prolactin and growth hormone (GH) who is treated successfully only with cabergoline as a first line treatment.

## 2. Case Presentation

A 46-year-old woman was admitted to our outpatient clinic with the complaint of swelling at her neck which she had become aware of recently. She had amenorrhea for fourteen years, as well as weight gain, frequent headaches, enlargement of the nose, increasing shoe size, and necessity to enlarge her rings within the past several years. She did not have galactorrhea or any visual symptoms. She had a history of hypertension for four years and dyslipidemia for one year. At her initial physical examination, thyroid gland was grade II palpable, and she had acral enlargement and central obesity. Depending on these findings, laboratory tests related to goiter and pituitary functions were performed. 

Her initial laboratory findings demonstrated hyperglycemia, hyperlipidemia, normal thyroid functions, low gonadotropin levels, and severe prolactin elevation with macroprolactin absence (serum prolactin > 40000 mIU/L, normal ranges: 33.36–580.80). The diagnosis of acromegaly was confirmed with elevated IGF-1 level (serum IGF-1: 550 ng/mL, normal ranges: 94–210) and failure to suppress GH levels with a 100 g oral glucose load. Accompanying Cushing's disease was excluded with a low-dose dexamethasone suppression test.

**Figure 1:**
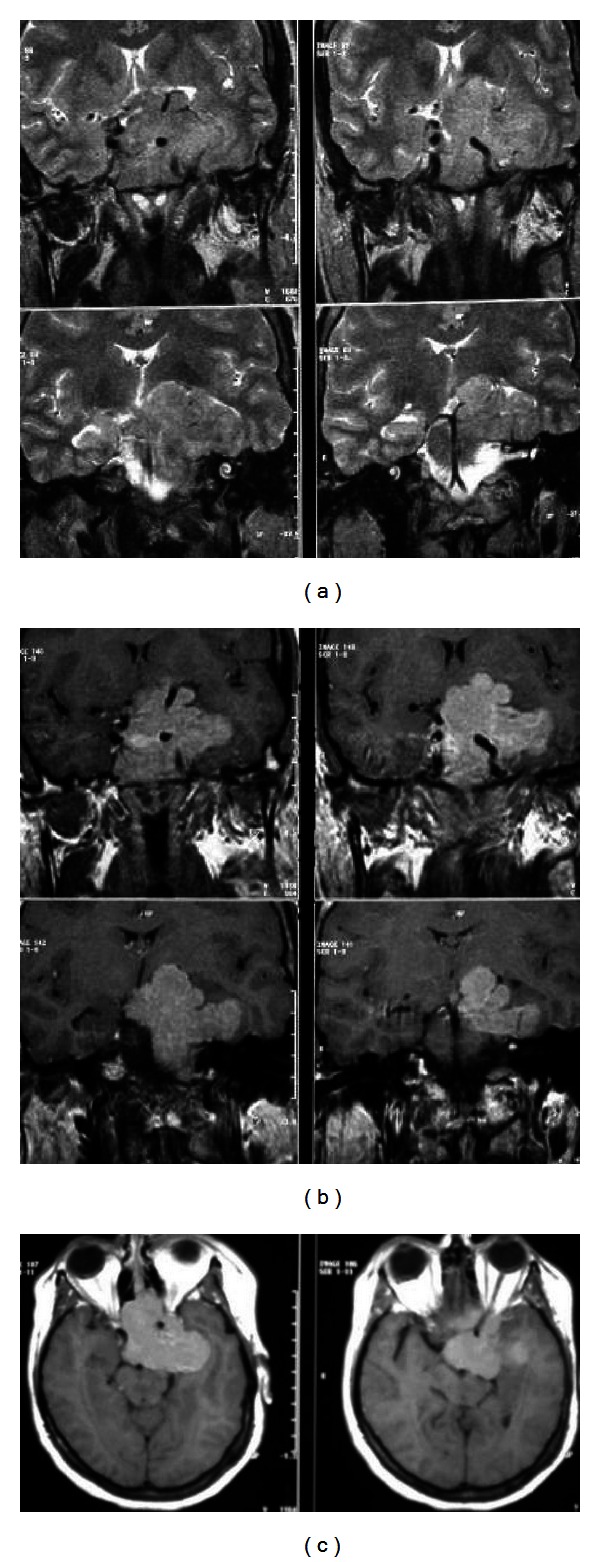
(a) Coronal T2-weighted turbo spin echo magnetic resonance image shows solid macroadenoma filling the entire sella, invading left cavernous sinus, and obliterating the suprasellar cistern; (b)-(c) postcontrast coronal (b) and axial (c) spin echo images show that the mass enhances intensely and homogenously. The extention of the macroadenoma in the prepontine cistern and the encasement of cavernous portion of the left internal carotid artery were more prominent in these images.

Her thyroid ultrasonography revealed multinodular goiter with the largest nodule exhibiting 15 mm diameter. Magnetic resonance imaging (MRI) of the sella showed a pituitary macroadenoma which has maximum diameters of 6 × 5 × 5.5 cm extending to the sphenoid sinus via clival destruction, suprasellar cistern, and prepontine cistern. Left cavernous sinus, left middle cranial fossa, and left orbital apex were invaded, wherein optic chiasm and circle of the Willis were elevated. The macroadenoma was encasing the cavernous portion of the left internal carotid artery (Figure 1). A minimal central scotoma in the right eye was found when her visual field was evaluated. After all, the patient was consulted to neurosurgeons and no emergent surgery was indicated.

Cabergoline treatment with a dosage of 2 mg per week was prescribed to the patient, as well as appropriate medical therapy for diabetes mellitus and hyperlipidemia. The patient was warned carefully about the symptoms of a possible pituitary apoplexy crisis. No side effects related to cabergoline treatment were observed during the follow-up period.

**Figure 2:**
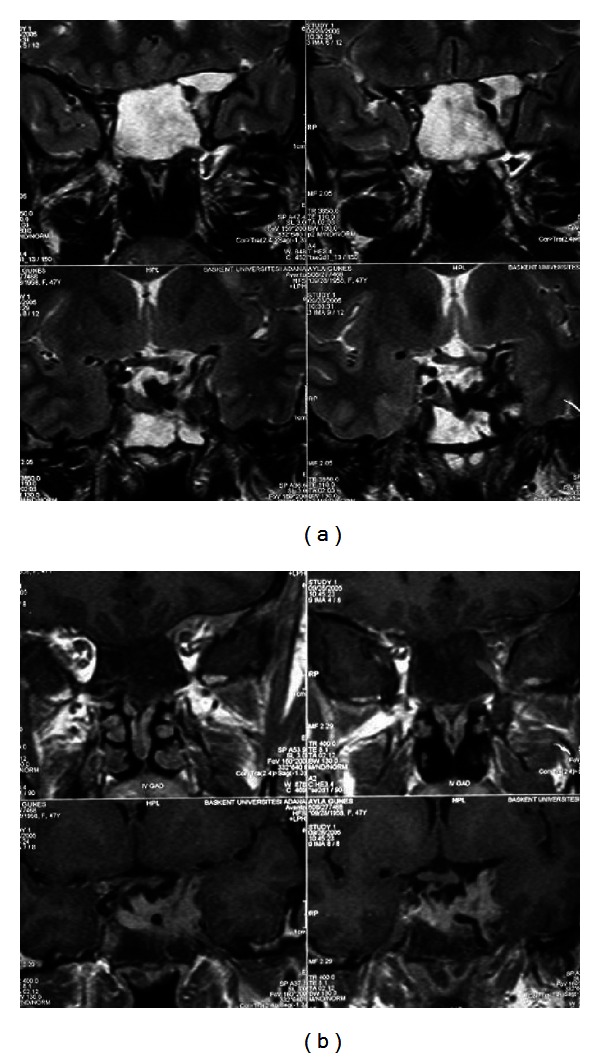
Follow-up magnetic resonance imaging, (a) coronal T2-weighted turbo spin echo image shows cyst formation within the macroadenoma with a small solid portion on the left lateral side. (b) Postcontrast coronal spin echo image revealed resolution of the macroadenoma in the suprasellar cistern.

After two months of therapy, her prolactin levels decreased to normal ranges (174.49 mIU/L) and the slight visual impairment in the right eye completely disappeared. Follow-up MRI study after 4 months of medical treatment showed prominent regression of the macroadenoma and presenting cystic degenerations, with a size of 3.5 × 2.5 × 2 cm. Suprasellar cistern was almost clear and optic chiasm was in normal location. There was a clear border between macroadenoma and adjacent left temporal lobe parenchyma (Figure 2). She lost 12.5 kg weight and her menstrual activity returned to normal at the fifth month of therapy. Her IGF-1 level decreased to 316 ng/mL and GH suppression was achieved with 100 g oral glucose load.

## 3. Discussion

Although most of the pituitary tumors are microadenomas some grow aggressively invading the extrasellar tissues. Various classifications have been proposed but there is no evident terminology to describe large pituitary tumors [13]. Despite the histological benign nature, the management of these adenomas is very difficult [5]. In the present case, we describe a rare pituitary adenoma secreting prolactin and GH synchronously which can be accepted as “giant.” Such a large pituitary adenoma is expected to cause distinct symptoms; however the patient's only complaint on admission was swelling at the neck. This implies surely that the patients examined in endocrinology clinics should be investigated thoroughly.

This patient has not undergone surgery yet, so the exact histology of the tumor is unknown. Nevertheless, according to the extensive spreading of the tumor and hyperprolactinemia dominancy, an acidophilic stem cell adenoma may be responsible for this phenomenon [14]. The nature of high proliferation and aggressive tumor growth should be kept in mind with such type of adenoma. Surgery should be considered for indicated patients.

It is well known that the majority of prolactinomas respond to dopamine agonists with a rapid lowering of prolactin. In addition to this effect, bromocriptine and cabergoline are capable of inducing significant tumor shrinkage within the first three months of therapy in the majority of tumors [6, 15]. Also as Sandret et al. reported in their meta-analysis, cabergoline single-agent therapy can normalize IGF-1 levels in one-third of patients with acromegaly [12]. They also reported that this effect may occur even in normoprolactinemic patients. From this point of view, our patient's response to cabergoline treatment is as expected. Moreover, cabergoline was capable of controlling acromegaly in short term for our subject. Beyond the treatment success in terms of biochemical and radiologic imaging findings, the progress of clinical findings was also satisfactory. Surely, accompanying metformin therapy for diabetes mellitus and life style changes had additive effects concerning the wonderful weight loss, but cabergoline contribution by prolactin reduction cannot be denied.

 In conclusion, our case suggests that cabergoline may be effective and safe as a first line monotherapy in patients with giant pituitary adenomas cosecreting prolactin and GH. However appropriate followups and changes in treatment plan must be made in long term for such patients.
